# Artificial Intelligence‐Based Approaches for AAV Vector Engineering

**DOI:** 10.1002/advs.202411062

**Published:** 2025-02-11

**Authors:** Fangzhi Tan, Yue Dong, Jieyu Qi, Wenwu Yu, Renjie Chai

**Affiliations:** ^1^ State Key Laboratory of Digital Medical Engineering Department of Otolaryngology Head and Neck Surgery Zhongda Hospital School of Life Sciences and Technology School of Medicine Advanced Institute for Life and Health Jiangsu Province High‐Tech Key Laboratory for Bio‐Medical Research Southeast University Nanjing 210096 China; ^2^ Immunowake, Inc. Shanghai 201210 China; ^3^ Department of Neurology Aerospace Center Hospital School of Life Science Beijing Institute of Technology Beijing 100081 China; ^4^ State Key Laboratory of Hearing and Balance Science Beijing Institute of Technology Beijing 100081 China; ^5^ School of Medical Engineering Affiliated Zhuhai People's Hospital Beijing Institute of Technology Zhuhai 519088 China; ^6^ Advanced Technology Research Institute Beijing Institute of Technology Jinan 250300 China; ^7^ School of Mathematics Southeast University Nanjing 210096 China; ^8^ Co‐Innovation Center of Neuroregeneration Nantong University Nantong 226001 China; ^9^ Department of Otolaryngology Head and Neck Surgery Sichuan Provincial People's Hospital School of Medicine University of Electronic Science and Technology of China Chengdu 610072 China; ^10^ Southeast University Shenzhen Research Institute Shenzhen 518063 China

**Keywords:** AAV vector engineering, artificial Intelligence, immunogenicity, transduction efficiency

## Abstract

Adeno‐associated virus (AAV) has emerged as a leading vector for gene therapy due to its broad host range, low pathogenicity, and ability to facilitate long‐term gene expression. However, AAV vectors face limitations, including immunogenicity and insufficient targeting specificity. To enhance the efficacy of gene therapy, researchers have been modifying the AAV vector using various methods. Traditional experimental approaches for optimizing AAV vector are often time‐consuming, resource‐intensive, and difficult to replicate. The advancement of artificial intelligence (AI), particularly machine learning, offers significant potential to accelerate capsid optimization while reducing development time and manufacturing costs. This review compares traditional and AI‐based methods of AAV vector engineering and highlights recent research in AAV engineering using AI algorithms.

## Graphical abstract

Adeno‐associated virus (AAV) has emerged as a leading vector for gene therapy. However, AAV vectors face limitations, including immunogenicity and insufficient targeting specificity. Various methods have been used to engineer the AAV vector. This review compares traditional and AI‐based methods of AAV vector engineering and highlights recent research in AAV engineering using AI algorithms.

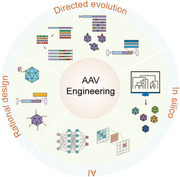

## 1 A Brief Introduction to the AAV Vector

AAV is a small, non‐enveloped virus first discovered in 1965 as a contaminant in adenovirus preparations from humans and monkeys.^[^ ^1^ ^]^ Since its isolation, AAV has become a promising vector for gene therapy because of its safety, low immunogenicity, broad tissue tropism, and ability for long‐term gene expression.^[^ ^2^ ^]^ AAV has been used to treat various genetic disorders. As of December 24, 2024, the US FDA has approved seven AAV‐mediated gene therapy drugs, and ≈331 AAV gene therapy clinical trials are listed on clinicaltrials.gov, accounting for ≈12% of all gene therapies.

AAV has a diameter of 25 nm and primarily consists of a genomic DNA and a capsid. The capsid is an icosahedral structure composed of 60 protein subunits, namely VP1, VP2, and VP3, at a ratio of 1:1:10.^[^ ^3^, ^4^ ^]^ These capsid proteins are crucial for the stability of viral particles and for the infection process because they determine the specificity of interactions with host cell surface receptors through their 3D structures.

**Figure 1:**
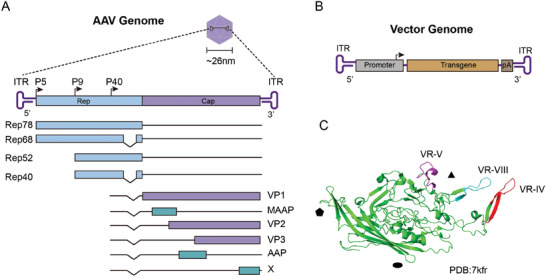
Genomic organization of AAV. A) The AAV capsid contains a ∼4.7 kb single‐stranded DNA genome, which is flanked by ITRs at both ends. Expression of two rep transcripts is driven by the p5 and p19 promoters, which undergo alternative splicing to ultimately produce four proteins (Rep78, Rep68, Rep52, and Rep40). The expression of the cap transcript is governed by the p40 promoter, leading to the synthesis of three capsid proteins (VP1, VP2, and VP3) along with assembly‐activating protein (AAP), membrane‐associated accessory protein (MAAP), and protein X. B) The schematic diagram of the AAV vector. C) Ribbon diagram of the AAV‐DJ VP3 structure. The inner surface of the capsid subunit is composed of jelly‐roll β‐barrel strands, with VR‐IV, VR‐V, and VR‐VIII distinctly highlighted in red, magenta, and cyan, respectively. These elements contribute to the formation of prominent spikes encircling the 3‐fold axis of the capsid.

The AAV genome is composed of a single‐stranded DNA (ssDNA) molecule ≈4.7 kilobases in length, which encodes the replication (rep) and capsid (cap) genes (**Figure** **1**A). The genome is flanked by inverted terminal repeats (ITRs) that form a T‐shaped hairpin structure.^[^ ^5^ ^]^ Specifically, the rep genes are located in the 5′ half of the genome, while the cap genes occupy the 3′ half. Each gene is transcribed from its respective promoter p5, p19, and p40. The rep region encodes four Rep proteins, Rep40, Rep52, Rep68, and Rep78, which play critical roles in viral replication, packaging, and transcriptional regulation. Notably, Rep78 and Rep68 are primarily responsible for resolving the covalently closed ends of the ITRs during viral DNA replication. These proteins exhibit helicase and endonuclease activities and bind to the Rep binding site within the ITRs, facilitating local denaturation. In contrast, Rep52 and Rep40, which possess 3′ to 5′ helicase activity, are essential for the encapsulation of viral DNA.^[^ ^6^, ^7^ ^]^ At the 3′ end of the viral genome, a single open reading frame encodes three capsid proteins, VP1, VP2, and VP3. Additionally, the cap coding region includes a 119 amino acid minor assembly‐activating protein,^[^ ^8^ ^]^ a 204 amino acid assembly‐activating protein,^[^ ^9^ ^]^ and a 172 amino acid X protein.^[^ ^10^ ^]^ Importantly, only the ITRs are essential for the propagation of recombinant AAV.^[^ ^11^ ^]^ As a result, the rep and cap genes, which comprise ≈96% of the AAV genome, can be substituted with an expression cassette containing a promoter, a therapeutic transgene, and a poly(A) tail (Figure 1B).

Due to its ssDNA genome, conventional AAV requires a transcription process to generate double‐stranded DNA (dsDNA) in cells, which leads to delayed gene expression. To enhance the rate of gene expression, self‐complementary AAV (scAAV) has been engineered to fold into dsDNA without DNA synthesis.^[^ ^12^ ^]^ As a result, scAAV typically demonstrates higher levels of gene expression compared to conventional single strand AAV (ssAAV), making it particularly suited for applications that demand rapid and high transgene expression.

Different AAV serotypes arise from variations in their capsid protein coding regions, leading to differences in tissue affinity and transduction efficiency.^[^ ^13^ ^]^ Mutations in these capsid proteins have resulted in the emergence of distinct serotypes, which can be identified through serological testing. A novel AAV serotype is defined by significant amino acid differences in the capsid protein gene sequences, along with its unique immunogenic properties and transduction characteristics. Each AAV serotype targets distinct receptors and exhibits unique tissue tropisms.^[^ ^13^ ^]^ Notably, AAV2 is considered the canonical serotype of the AAV family and serves as a valuable reference for the study of other AAVs. The structures of some natural AAV serotypes have been fully solved,^[^ ^14^, ^15^, ^16^, ^17^, ^18^, ^19^, ^20^, ^21^, ^22^, ^23^, ^24^, ^25^ ^]^ providing high‐resolution insights into the evolution of virus capsids. These structural analyses have enhanced our understanding of the mechanisms underlying capsid diversity and function.

Natural AAV capsids often exhibit insufficient cell or tissue specificity, which limits their applicability in precision medicine. Engineered AAV vectors can deliver transgenes into a wide range of dividing and non‐dividing cell types with higher efficiency and specificity. In dividing cells, transgene expression is gradually reduced, primarily due to cell division. In contract, AAV may enable stable expression in non‐dividing cells. However, factors such as the host immune response to foreign DNA,^[^ ^26^ ^]^ and the epigenetic state of the cell^[^ ^27^, ^28^ ^]^ may limit the expression of the transgene. Moreover, AAV has a dilution effect because of the neutralization of capsid by pre‐existing antibodies and the presence of the inner limiting structures.^[^ ^29^ ^]^ Multiple times of AAV injection is a strategy to main the transgene expression, but high‐titer neutralizing antibodies (NAbs) dilute the effect. Several strategies, including plasma exchange.^[^ ^30^ ^]^ and the use of empty capsids as decoys,^[^ ^31^ ^]^ have been developed to address this problem. Recently, a study used imlifidase (IdeS), a streptococcal cysteine protease, which can cleave IgG to eliminate anti‐AAV antibodies.^[^ ^32^ ^]^ In mice and nonhuman primates that were passively immunized, administration of IdeS reduced anti‐AAV antibody levels and facilitated efficient liver gene transfer of AAVs again. Furthermore, surface encapsulation or tethering using lipids, hydrogels, and polymers can efficiently protect the AAV capsids from NAbs, enabling them to evade detection and antibody responses.^[^ ^33^ ^]^ An alternative strategy is to engineer the AAV capsid to remove the epitopes that interact with NAbs, potentially enhancing the effectiveness of repeated AAV administration.

Through capsid and DNA element engineering, novel AAV vectors can be designed to more accurately target specific cell types or diseased tissues, enhancing and maintaining the therapeutic efficacy while minimizing effects on healthy cells. Additionally, AAV vectors are widely known for their low immunogenicity. However, they can trigger dose‐dependent immune responses.^[^ ^34^ ^]^ Engineering AAV vectors can help mitigate these immune responses,^[^ ^35^ ^]^ thereby enhancing their safety.

## 2 AAV Capsid Engineering

The AAV capsid exhibits an icosahedral structure with two‐fold, three‐fold, and five‐fold symmetry axes.^[^ ^16^ ^]^ Prominent spike structures containing the variable regions (VRs) are situated on the three‐fold symmetry axes,^[^ ^6^ ^]^ and these play a crucial role in determining the capsid's tissue specificity and immunogenicity. Due to their exposed positions, these protrusions are ideal sites for peptide insertion to enhance receptor binding and modify vector tropism. Each capsid subunit contains nine variable regions (VRs) on the surface of the virion, which exhibit significant sequence diversity, particularly in VR‐IV, VR‐V, and VR‐VIII^[^ ^36^ ^]^ (Figure 1C). VR‐IV and VR‐VIII loops have consistently been targeted as insertion sites for peptides insertion.^[^ ^37^ ^]^ These loops are critical for mediating virus‐receptor interactions. Researchers often use saturation mutagenesis to create diverse AAV libraries targeting these regions, allowing for modulation of the interactions between the virus and host cell surface receptors. For instance, the team led by Viviana Gradinaru developed a new variant, AAV.CAP‐B10, using AAV9 as a scaffold and employing directed evolution techniques.^[^ ^38^ ^]^ This variant integrates an additional targeting peptide at VR‐IV (the 455th amino acid of AAV9). After intravenous injection, AAV.CAP‐B10 can efficiently cross the blood‐brain barrier (BBB) to target neurons while avoiding accumulation in the liver, thereby preventing potential liver toxicity side effects. Additionally, specific phosphorylation sites on the capsid protein influence the virus's packaging, assembly, and infection efficiency,^[^ ^39^, ^40^, ^41^ ^]^ and optimizing these sites can lead to enhanced stability and delivery efficiency of the viral particles. Furthermore, the N‐termini of VP1 and VP2 contain unique sequences that play crucial roles in the virus's invasion and uncoating processes.^[^ ^42^, ^43^ ^]^ Modifications in these regions may influence the virus's infection efficiency and gene release.

**Figure 2:**
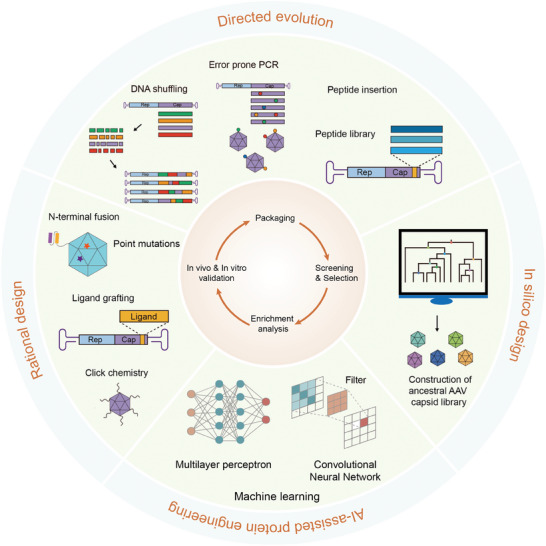
Methods of AAV capsid engineering. AAV capsids can be engineered to enhance or optimize their properties. Several strategies have been developed for this purpose, including rational design, directed evolution, in silico design, and AI‐assisted protein sequence design.

Through precise engineering of these key regions, researchers can design novel AAV capsid variants with improved yield, specificity, and ability to evade host immune responses along with increased stability and delivery efficiency. Common AAV capsid engineering techniques include directed evolution, rational design, in silico design, and AI‐assisted protein engineering (**Figure** **2**).

## 3 Directed Evolution

Directed evolution simulates the process of natural selection and can effectively optimize the functionality of AAV capsids without relying on prior knowledge of the capsid's structure or the mechanisms through which it is created. By establishing a library of AAV capsid mutants and using high‐throughput screening methods, variants of AAV capsids with specific biological characteristics can be selected. There are various commonly used directed evolution techniques (Figure 2). The error‐prone PCR method is often used to produce DNA sequences with random mutations to study protein function, structure, and interactions. This method increases the mutation rate by using a DNA polymerase with a high error rate, thereby creating a diverse DNA library for screening variants with specific properties.^[^ ^44^ ^]^ DNA‐shuffling technology is another method to create complex capsid libraries from different wild‐type viruses. By using this method, researchers have generated a new chimeric AAV, AAV‐DJ, that performs better than natural AAV serotypes in cell cultures and in mouse livers.^[^ ^45^ ^]^

Besides these two methods, the commonly used method is peptide insertion in the VR loop regions of the capsid (Figure 1C). Previous screening techniques using peptide insertion tended to focus on homogeneous cell types and to neglect cell specificity, but a recently developed method called CREATE allows for the screening of AAV capsids in specific cell populations in vivo.^[^ ^46^ ^]^ In this technique, an AAV library is constructed by inserting a random sequence of seven amino acids into the VR‐VIII loop of the AAV9 capsid. These variants are injected into GFAP‐Cre mice, and capsid sequences are selectively recovered by Cre‐mediated recombination and PCR. CREATE has developed an AAV variant, AAV‐PHP.B, that effectively and broadly transduces astrocytes and neurons in mouse brain following intravenous injection. Its gene transfer efficiency throughout the CNS is at least 40 times greater than that of AAV9. However, the CREATE method relies on the use of Cre transgenic animal models, and this limits its application, especially in non‐human primates. Compared to the methods described above, the combination of AAV library driven by specific promoters and RNA sequencing can generate new AAV variants with high efficiency and specificity in wild‐type animals. A muscle‐specific promoters mediated RNA screening strategy, named DELIVER, has been used to generate a group of novel AAV variants featuring RGD motifs in their capsids. These variants demonstrate superior efficiency and selectivity in transducing muscle tissue in mice and non‐human primates.^[^ ^47^ ^]^ Similarly, another RNA‐driven screening platform termed TRACER uses neural‐specific promoters,^[^ ^48^ ^]^ and a series of AAV variants targeting neurons with high efficiency were generated. Theoretically, new AAV variants with specificity to any different organs can be generated through specific promoter‐mediated AAV library screening.

Sometimes different library construction methods are used simultaneously. To develop an AAV capable of delivering genes to the outer retina, one study used three AAV libraries.^[^ ^49^ ^]^ The first library was inserting a random 7aa sequence into the VR loop of AAV2 capsid. The second library used a tyrosine mutant in AAV2 capsid (AAV2 Y444F) to perform random mutations. The third library was generated by DNA shuffling of capsid proteins from AAV1, 2, 4, 5, 6, 8, and 9. Ultimately, the AAV vector variant AAV2.7m8 was selected, successfully achieving gene delivery to retinal cells and showing the potential for treating inherited degenerative diseases of both the inner and outer retina.

In summary, the directed evolution process requires multiple iterations, each time selecting the best candidates for the next round of mutation and screening.^[^ ^50^, ^51^, ^52^, ^53^ ^]^ Researchers can progressively evolve AAV serotypes to achieve effective targeting of specific cell types. The choice of method may depend on the specific target cell type and the desired characteristics of the AAV variant. The overall timeframe for evolving AAV serotypes can range from several weeks to several months, depending on the complexity of the approach and the efficiency of the screening and validation processes.

## 4 Rational Design of AAV Capsids

Rational design has been used to generated AAV variants with low immunogenicity. Introducing point mutations in the epitopes of AAVs that are recognized by neutralizing antibodies (NAbs) may diminish recognition by host antibodies, thereby reducing immunogenicity and enhancing transduction efficiency.^[^ ^54^, ^55^ ^]^ There are three main methods used to map antigenic epitopes: directed evolution, which selects AAV variants with neutralizing epitope mutations; epitope scanning, involving peptide scanning, insertion, or site‐directed mutagenesis; and structural biology, utilizing 3D reconstruction of AAV capsid‐antibody fragment complexes.^[^ ^56^ ^]^ These approaches provide insights into the structure of AAV capsids and their interactions with host antibodies, informing the design of AAV vectors that evade neutralization. AAV1, AAV2, AAV5, and AAV8 have the epitope mapping,^[^ ^56^ ^]^ and CD8^+^ T‐cell epitopes are conserved between human and mice,^[^ ^57^, ^58^ ^]^ suggesting that AAVs with reduced NAb binding in mice may also work in humans.

There are also various strategies for rational design to enhance transduction efficiency. A chimeric AAV variant named AAV2.5 was created by introducing five mutations from AAV1 into the AAV2 capsid.^[^ ^59^ ^]^ These mutations are located in the VRs. AAV2.5 combines the muscle targeting capability of AAV1 while retaining the receptor binding ability of AAV2. The binding characteristics of AAV can be altered by simply grafting motifs involved in cell‐type‐specific receptor binding into different serotypes of AAV. For example, researchers have created new chimeric strains, AAV2G9 and AAV2i8G9, by transplanting the galactose binding footprint of AAV9 onto AAV2 and AAV2i8, and these new strains show enhanced transduction efficiency while maintaining tissue tropism.^[^ ^60^ ^]^

Functional peptide insertion is another efficient strategy to generate AAV variants. Our previous study developed a new AAV vector named AAV‐ie^[^ ^61^ ^]^ by inserting a peptide from AAV‐PHP.eB.^[^ ^62^ ^]^ into one of the VR loop of AAV‐DJ and found that this insertion could change the tropism of AAV‐DJ in the inner ear.

In the modification of AAV capsid proteins, click chemistry is also crucial for enhancing the functionality and application of viral vectors. Click chemistry can be used to precisely attach specific small molecules, peptides, or antibodies to the AAV capsid, thus enhancing the virus's tissue or cell specificity and modifying its epitopes to reduce recognition by NAbs.^[^ ^63^, ^64^ ^]^

Rational design offers the advantage of utilizing smaller and more targeted mutation libraries compared to directed evolution, thereby reducing screening efforts. However, the limited understanding of fundamental biological processes, such as protein folding and natural evolutionary mechanisms, poses significant challenges for the de novo design of AAV functionality using rational design methods. Additionally, rational design encounters difficulties in developing strategies for immune evasion. NAbs target both linear epitopes with continuous active sites and conformational epitopes with active sites distributed across different regions of the AAV capsid, thus complicating the rational alteration of these sites.^[^ ^65^ ^]^

## 5 In Silico AAV Capsid Design

In protein evolution, ancestral proteins generally possess characteristics such as thermal stability, making them ideal scaffolds for further directed evolution.^[^ ^66^ ^]^ These characteristics are closely related to several factors.^[^ ^66^ ^]^ First, environmental adaptability is crucial. The high‐temperature conditions of the Precambrian era likely drove proteins to evolve greater thermal stability. Second, the consensus effect is commonly observed when reconstructing ancestral proteins, significantly enhances their stability. This effect occurs when rare amino acids are replaced by more common ones, resulting in reconstructed proteins displaying unusually high thermal stability. Additionally, the high mutation rates and less efficient protein quality control systems in ancient organisms may have further contributed to the dynamic and thermodynamic stability of these proteins. Although ancestral proteins demonstrate high thermal stability and mutation resilience, they still need to evolve to adapt to the ever‐changing environment and biological requirements.

Ancestral Sequence Reconstruction (ASR) is a bioinformatics and phylogenetic method used to infer and reconstruct the gene or protein sequences of extinct or unknown ancestral organisms. This approach is based on genomic data from extant species and involves comparing sequence differences among various species to infer ancestral sequences using phylogenetic tree models and statistical methods.^[^ ^67^ ^]^ This method can also be used in the design of AAV capsids due to the sequence similarities among natural AAVs. For example, the homology among AAV1 to AAV10 ranges from ≈50% to 99%.^[^ ^25^ ^]^ Phylogenetic analysis reveals that AAV1 through AAV10 are distributed across six clades (A to F) and two clones, based on extensive sequencing and computational methods.^[^ ^25^, ^68^ ^]^ Researchers have generated functional putative ancestral AAVs by predicting evolutionary intermediates of capsids of natural AAVs, and one ancestral AAV, Anc80L65, has been identified as an efficient gene therapy vector for the liver, muscle, and retina.^[^ ^69^ ^]^ Another study employed computational design and experimental construction to create a library of putative ancestral AAVs, thus generating new capsid variants with potentially enhanced infectious capabilities.^[^ ^70^ ^]^ This study found that these ancestral AAVs had higher in vivo infection efficiency than modern serotypes, particularly showing superior performance in the gastrocnemius muscle of mice compared to the clinically used AAV1.

The design of AAV capsids through evolutionary methods depends on the phylogenetic analysis between AAV serotype. If the available modern sequence data is insufficient or the phylogenetic relationships are not fully understood, the inferred ancestral sequences may be inaccurate.

## 6 AI‐Assisted AAV Capsid Engineering

Machine learning (ML), a crucial subset of artificial intelligence (AI), enables computers to learn from data and make decisions using algorithms, eliminating the need for extensive programming,^[^ ^71^ ^]^ which is different from in silico models. In silico models are based on established scientific principles and mathematical equations, simulating biological processes to predict outcomes, often requiring detailed knowledge of the underlying mechanisms.^[^ ^67^, ^69^ ^]^ In contrast, ML uses large datasets to automatically identify patterns and make predictions without the need for explicit modeling, allowing for greater flexibility and adaptability.^[^ ^71^, ^72^ ^]^ The amount of data needed for ML can differ greatly based on the intricacy of the task at hand and the specific algorithm employed. High‐performance models typically necessitate datasets consisting of thousands to millions of samples to ensure robust generalization and accuracy. However, if the quality of the training data is poor, with inaccurate labels or systematic differences from the test data, even large datasets may result in suboptimal model performance in real‐world applications.^[^ ^72^ ^]^ This technology can process large volumes of biological data, quickly identifying promising capsid designs that enhance predicted AAV packaging efficiency and cell transduction efficiency. Consequently, ML not only accelerates the experimental process, but also facilitates the design of novel AAV capsids that may be challenging to conceive using traditional methods, ultimately improving therapeutic efficacy and safety. Although the development of AI technology has advanced rapidly, its application in AAV engineering is still in its early stages. Currently, only a limited number of studies have leveraged ML for engineering AAV vectors, and these have primarily focused on capsids and promoters (**Table** **1**).

**Table 1:** List of papers about AAV capsid and gene element engineering using ML methods.

Year of publication	Modification site	Machine learning methods	Function	DOI
2019	AAV2 capsid	Boltzmann distribution, PCA	Liver tropism	https://doi.org/10.1126/science.aaw2900
2021	AAV2 capsid	ANN, SVM	Viral assembly prediction	https://doi.org/10.1016/j.omtm.2020.11.017
2021	perspective article	Supervised and unsupervised models	Immune evasion	https://doi.org/10.3389/fimmu.2021.674021
2021	AAV2 capsid	LR, CNN, RNN	Multiple tissue tropism	https://doi.org/10.1038/s41587‐020‐00793‐4
2023	AAV2 capsid	Transformer, CNN	CNS tropism	https://doi.org/10.3390/v15040848
2023	AAV2 capsid	SVAE	BBB tropism	https://doi.org/10.1371/journal.pbio.3002112
2024	AAV9 capsid	Fit4Function	Liver tropism	https://doi.org/10.1038/s41467‐024‐50555‐y
2024	AAV2 capsid	Biophysically‐inspired model	Predict and select viable AAV2 variants	https://doi.org/10.1186/s12859‐024‐05823‐5
2024	AAV5 capsid	Linear models and neural networks	Human brain tropism	https://doi.org/10.1126/sciadv.adj3786
2019	Promoter	DeePromoter (CNN, LSTM)	Promoter prediction	https://doi.org/10.3389/fgene.2019.00286
2023	Promoter	PromGER model	Promoter prediction	https://doi.org/10.3390/genes14071441
2023	Promoter	EVMP framework	Synthetic promoter strength prediction	https://doi.org/10.3389/fmicb.2023.1215609
2024	Promoter	Puffin model	Transcription initiation prediction	https://doi.org/10.1126/science.adj0116
2024	Promoter	CAPE framework	Promoter evolution	https://doi.org/10.1093/bib/bbae398
2022	Enhancer	LR, SVM, CNN, Split CNN, VGG, RNN,CrepHAN	Enhancer prediction	https://doi.org/10.1016/j.ygeno.2022.110454
2023	Enhancer	TACIT (CNN)	Enhancer activity prediction	https://doi.org/10.1126/science.abm7993

**Abbreviation**: PCA: Principal Component Analysis; ANN: Artificial Neural Network; SVM: Support Vector Machine; LR: Logistic Regression; CNN: Convolutional Neural Network; RNN: Recurrent Neural Network; SVAE: Supervised Variational Autoencoder; LSTM: Long Short‐Term Memory; EVMP: Extended Vision Mutant Priority; CAPE: Chaos‐Attention net for Promoter Evolution; VGG: Visual Geometry Group; CrepHAN: cross‐species enhancer predictor based on a hierarchical attention network; TACIT: Tissue‐Aware Conservation Inference Toolkit.

The integration of deep sequencing or next‐generation sequencing with ML methods enables the development of novel functional AAV capsids. Next‐generation sequencing efficiently generates large volumes of data, supplying ML algorithms with extensive datasets to analyze genetic variations and diversity in AAV capsids, thereby identifying beneficial genetic mutations that enhance capsid functions.^[^ ^73^ ^]^ Through precise mutation analysis, researchers can pinpoint specific genetic changes in the AAV capsid DNA sequence, allowing AI models to better understand the impact of these mutations on capsid performance.^[^ ^8^ ^]^ Furthermore, when combined with ML, deep sequencing can screen for capsid variants with desirable biological traits, such as improved tissue specificity, enhanced gene delivery capabilities, or the ability to evade the immune response.^[^ ^74^, ^75^, ^76^ ^]^ Deep sequencing also provides detailed data for training and validating ML models, and experimental validation of predicted outcomes further optimizes the accuracy and reliability of these models.^[^ ^71^ ^]^ Additionally, the synergy between deep sequencing and machine learning presents promising opportunities for the identification of common motifs that enhance the functionality of AAV capsids.

In the process of AAV engineering, although a vast library of AAV variants has been generated through mutagenesis, the efficiency of these variants successfully assembling into AAV virions remains low.^[^ ^73^, ^77^ ^]^ Enhancing AAV assembly efficiency ensures that more functional viral particles are successfully assembled and utilized for gene delivery, which in turn helps reduce production costs and makes gene therapy more economically feasible. ML plays a significant role in predicting AAV assembly. By utilizing data from the AAV capsid library before and after virus assembly, Marques et al. used ML algorithms such as artificial neural networks (ANN) and support vector machines (SVM) to train models that predict whether unknown AAV capsid protein variants can assemble into viable virus‐like structures.^[^ ^77^ ^]^ That study identified specific amino acid residues that are crucial for the AAV2‐derived capsid proteins and demonstrated the predictive capability of ML in viral vector design through the generation of comparative libraries. In this paper, ANN and SVM exhibit different performances in predicting AAV virus assembly capabilities. In terms of accuracy, the optimized ANN model achieves a prediction accuracy of 68.18%, while the SVM model slightly outperforms ANN in this aspect. Additionally, the two models differ in their reliance on data representation. The SVM model, after testing various kernel functions (linear, polynomial, RBF, and sigmoidal kernels) and two data representation methods (amino acid residue representation and physicochemical property representation), found that the combination of the RBF kernel and amino acid residue representation yielded the best results. In contrast, the ANN model demonstrates greater robustness, achieving good predictive performance across different hyperparameter settings (such as node count and learning rate). While the SVM model slightly surpasses the ANN model in prediction accuracy, the ANN model is more robust and less sensitive to hyperparameter selection. Both models show promising performance when handling this type of biological data. Leonardis et al. performed an unsupervised modeling study of the viability mutational landscape of AAV2, focusing on the capsid region. Their research aimed to predict the feasibility of genetic variations in deep mutational scanning experiments using a biophysically inspired model.^[^ ^78^ ^]^ The model is trained on different datasets to explore various aspects of the mutation landscape influenced by the selection process. Key findings include the development of a statistical energy model as a representative of sequence vitality and the successful application of the model for classifying sequences as viable or non‐viable. Their study highlights the importance of combining high‐throughput experimental techniques with ML to enhance predictions of how genetic variations will affect capsid vitality.

ML models that are trained directly on experimental data have the capability to fully explore the diverse potential of engineered proteins. Bryant et al. successfully designed a large number of highly diverse AAV2 capsid protein variants using ML methods such as recurrent neural networks (RNN), convolutional neural networks (CNN), and logistic regression (LR) that all maintained the capability to package DNA payloads.^[^ ^73^ ^]^ But CNN and RNN trained on experimental data outperform LR models in generating highly diverse AAV2 capsid variants. Additionally, CNN and RNN models show more robustness across different training datasets, whereas the performance of the LR model is significantly affected by the size of the training data. The research team generated 201426 variants of the AAV2 wild‐type sequence, of which more than 110000 were viable, with 57348 exhibiting diversity that surpassed the average levels of natural AAV serotypes. Even with limited training data, deep neural network models can accurately predict the viability of these highly diverse variants. This method unlocks extensive regions of functional sequence space that were previously unreachable, thereby offering new opportunities for the creation of enhanced viral vectors.

The modification of AAV capsids is crucial for immune evasion, reducing patient immune responses, and enhancing the safety and efficacy of AAV‐based therapies. Engineered capsids can evade pre‐existing antibodies and immune detection, thus enabling repeated administrations and broader patient applicability. This is particularly important for the successful delivery of gene therapies, where immune responses can otherwise limit therapeutic potential and longevity. A study by Sinai et al. focused on a 28‐amino acid segment of the AAV2 capsid, and they successfully generated viable variants with multiple mutations through interpolation in the sequence space using a variational autoencoder (VAE), thus generating diverse and novel capsids capable of immune evasion.^[^ ^74^ ^]^ Their study utilized multiple sequence alignment data from 564 samples of dependoparvoviruses to train VAE and 22704 samples from deep mutational exploration on the target region to further enrich the dataset, resulting in a variety of capsids potentially optimized for gene delivery. This approach not only highlights the importance of protein engineering in therapy, but also demonstrates the significant potential of computational methods like VAE for diversifying and optimizing protein structures for specific functions, thus providing multiple potential choices for optimizing gene delivery.

ML has also been used to improve the delivery and transduction efficiency of AAVs. Eid et al. developed a novel method called Fit4Function for systematically designing multi‐trait AAV capsids.^[^ ^79^ ^]^ Fit4Function creates a capsid library to evenly sample the sequence space, thereby acquiring consistent and reliable screening data to develop precise sequence‐to‐function models. Their study successfully designed a multi‐trait capsid library and validated 89% of the library variants across all six predefined standards. The combination of six models accurately predicted the biological distribution of AAV capsid mutations in macaques, leading to higher production, enhanced transduction efficiency in mouse liver cells and human hepatocyte compared to AAV9. The Fit4Function strategy can predict cross‐species traits of modified AAV capsids, thus aiding in developing an ML map for predicting various traits related to AAV capsid performance. Han et al. used computer‐aided directed evolution to create novel AAV variants that exhibit high transduction efficiency in the brain.^[^ ^75^ ^]^ Their study focused on the VR‐IV region of the AAV capsid protein, specifically targeting amino acid positions 442–469. By training deep learning models on previous datasets, the researchers generated a highly diverse library of ∼95089 viral vectors and identified two new AAV variants, AAV2.A1 and AAV2.A2, with significantly higher transduction efficiency in the central nervous system compared to AAV2. This work demonstrates the potential of computer‐aided directed evolution for enhancing the application of AAV vectors for gene therapy.

Modifying AAVs to enable them to cross the BBB holds significant promise for application in neurological diseases, and a recent study described the engineering of peptide‐modified AAV capsid proteins that enable new interactions with LY6A or LY6C1 proteins, which are expressed on the BBB in mice, thus achieving targeting of the central nervous system.^[^ ^76^ ^]^ The researchers used a supervised VAE (SVAE) model for ML training and successfully generated thousands of capsids with significantly enhanced central nervous system targeting through in vitro screening and in vivo validation methods. The SVAE model consists of three neural network modules: an encoder (X→Z), a decoder (Z→X), and a regressor ((X, Z)→Y). The encoder and decoder together form a standard VAE, while the regressor is trained using supervised information. The encoder encodes an input 7‐amino acid sequence (X) into a 2D latent space representation (Z). The decoder reconstructs the original sequence from the latent space. The regressor predicts the log2 enrichment value (Y) of the sequence based on both the sequence (X) and its latent representation (Z). During training, the encoder and decoder networks are jointly trained with the objective of minimizing reconstruction loss. In contrast, the regressor is trained separately, aiming to minimize prediction loss. Additionally, there is a distribution loss term that constrains the latent space to follow a standard Gaussian distribution. The SVAE model learns the mapping from sequences to functions through encoding‐decoding and supervised regression, enabling effective predictions of the performance of new sequences. Through the training and prediction capabilities of the SVAE model, researchers can rapidly screen a series of efficient AAV capsid variants targeting the central nervous system. This approach not only minimizes the reliance on animal experiments but also offers reliable and quantitative data for subsequent saturation mutagenesis and ML‐guided exploration of the capsid sequence space.

In the engineering modification of AAV capsids, various AI models exhibit different working principles and application potentials. RNNs are highly effective for handling sequential data, enabling the capture of temporal or spatial dependencies within sequences.^[^ ^71^ ^]^ RNNs can be utilized to predict the functional and structural characteristics of amino acid sequences. Although CNNs excel at handling image data, they can also be used for sequence data by extracting local features using convolutional layers.^[^ ^72^ ^]^ CNNs are capable of identifying feature patterns within sequences, such as conserved regions and mutation sites. VAEs are generative models that can learn the distribution of latent space, making them suitable for generating new variants.^[^ ^74^ ^]^ VAEs can be employed to generate variants with specific functionalities, thereby exploring the variant space. Generative adversarial networks (GANs) produce high‐quality samples through adversarial training.^[^ ^80^, ^81^ ^]^ GANs can be used to generate new viral variants, particularly when it is necessary to create variants that are similar to existing ones but possess different characteristics. In contrast, LR, as a simple and efficient linear model, is suitable for predicting the functional characteristics of specific variants, but it has limited capability in handling complex nonlinear relationships.^[^ ^72^ ^]^ Therefore, in the engineering modification of AAV capsids, the choice of an appropriate model depends on the specific research objectives and data characteristics.

In conclusion, a variety of methods have been employed to engineer AAV capsid, each with its own set of advantages and disadvantages (**Table** **2**). The combination of deep sequencing and ML offers unique benefits, making the selection of the appropriate algorithm critical for model construction based on different datasets. Achieving the optimal model requires the exploration of multiple algorithms and continuous parameter tuning. This iterative process not only improves prediction accuracy, but also enhances our understanding of the biological mechanisms underlying AAV capsid optimization. Ultimately, a comprehensive strategy that incorporates multiple algorithms may yield the most effective solutions to complex biological challenges.

**Table 2:** Comparison of AAV capsid engineering methods.

Technique	Advantages	Drawbacks
Rational design	‐ Small library size ‐ Precise modifications based on known structural and functional data ‐ Can design solutions for specific biological problems	‐ Low diversity ‐ Requires detailed structural and functional information, limited ability to predict effects on unknown areas ‐ Success rate may be limited by existing biological and structural knowledge
Direct evolution	‐ Large library size ‐ Large diversity of mutants ‐ Can discover new functional variants without detailed molecular mechanism knowledge ‐ Can explore a vast sequence space, discovering unexpected beneficial mutations	‐ Screening process may be time‐consuming and costly ‐ May produce a large number of irrelevant variants, requiring effective screening strategies
In silico design	‐ By restoring ancient protein forms, potentially unique advantageous capsid variants can be discovered ‐ Can explore potential evolutionary paths of proteins, providing guidance for future designs	‐ Reconstruction of ancient sequences is based on assumptions and may not be entirely accurate ‐ Compatibility with cells might be an issue
Machine Learning	‐ Can handle large amounts of data and can identify complex patterns and relationships and optimize design strategies ‐ Predictive accuracy and applicability will continue to improve with better data and algorithms	‐ Requires a large amount of training data, and results depend on the quality and coverage of data ‐ Interpretability may be poor, making it difficult to understand why the model makes specific predictions

## 7 AAV Regulatory Element Engineering

In gene therapy using AAV vectors, the choice of promoter is crucial for the spatiotemporal specificity of gene expression. Statistics show that the most commonly used promoters in clinical applications are ubiquitous promoters, such as CBA, CAG, and CMV.^[^ ^82^ ^]^ 45% of clinical trials involving publicly available promoters selected one of these three. However, when we focus on recent clinical trials, we can observe a new trend in promoter usage, with more researchers shifting their attention to the selection of tissue‐specific promoters. Over 25 clinical trials have utilized strong tissue‐specific promoters, such as Albumin and Synapsin, to achieve gene expression in specific tissues.^[^ ^82^ ^]^ By introducing cell and tissue‐specific gene regulatory elements, such as promoters and enhancers, the expression specificity of AAV vectors can be significantly enhanced.^[^ ^83^ ^]^ However, the natural promoters may not provide sufficient expression efficiency and specificity in certain cell types, thus limiting their therapeutic applications. Additionally, most natural gene regulatory element sequences are relatively long and cannot be packaged into the 4.7 kb AAV genome. Due to the limited understanding of these large endogenous elements, truncation may compromise their functionality. To address this issue, scientists have proposed generating and screening large synthetic promoter libraries that offer an ideal alternative for the engineering of AAV promoters.^[^ ^84^ ^]^

Furthermore, the screening process of synthetic promoter libraries can be integrated with modern bioinformatics and ML technologies,^[^ ^85^, ^86^, ^87^, ^88^, ^89^, ^90^, ^91^ ^]^ further improving the efficiency and accuracy of the screening. For example, Yang et al. proposed a universal framework called EVMP to enhance the performance of ML models in the task of synthetic promoter strength prediction.^[^ ^91^ ^]^ EVMP converts synthetic promoter sequences into base promoters and corresponding k‐mer mutations, which are then processed separately by BaseEncoder and VarEncoder, allowing for better utilization of mutation information. Additionally, EVMP provides an optional data augmentation method that generates multiple copies of the same synthetic promoter by selecting different base promoters, further improving model performance. Experimental results indicate that the EVMP framework significantly reduces the dependence on high‐quality annotated data while maintaining the same prediction accuracy. Compared to existing state‐of‐the‐art methods, EVMP improves the MAE and R^2^ metrics by 15.25% and 4.03%, respectively. EVMP is a robust and universal ML enhancement framework that holds promise for application in other mutation‐based synthetic biology component library tasks.

Additionally, a deep learning model called DeePromoter integrates CNN and Long Short‐Term Memory (LSTM) networks to accurately identify promoter sequences in both humans and mice.^[^ ^88^ ^]^ The CNN component is composed of multiple parallel convolutional layers specifically designed to learn critical motifs from the input sequences, utilizing convolutional kernels of varying sizes (27, 14, and 7). In contrast, the LSTM component captures the long‐term dependencies among these motifs, enhancing the network's depth while keeping the number of parameters relatively low. The outputs from the CNN and LSTM are concatenated and subsequently processed through a fully connected layer for final classification predictions. This innovative combination of CNN and LSTM effectively extracts complex features, achieving high prediction accuracy on a challenging negative set that includes difficult samples, thus outperforming methods that rely solely on CNN or LSTM. A significant advancement of this model is its use of a more challenging negative set derived from promoter sequences rather than non‐promoter regions, which greatly enhances the model's discrimination capability and reduces false positive predictions.

Utilizing ML and high‐throughput sequencing for the screening of synthetic promoter libraries provides a revolutionary method for the engineering of AAV promoters in gene therapy. First, a diverse and functionally relevant synthetic promoter library is generated. Next, high‐throughput sequencing technology is applied to rapidly and comprehensively assess the expression levels of these promoters in different cell types, thus generating extensive amounts of data. Subsequently, ML techniques are employed to analyze and model this data, identifying key sequence features associated with efficient and specific expression. Through this integrated approach, not only can the screening process for high‐performance promoters be significantly accelerated, but potential high‐quality promoter sequences that are difficult to identify using traditional methods can also be identified.

The engineering of AAV regulatory elements is a critical step in the development of effective gene therapies. Utilizing databases such as UCSC Genome Browser, Ensembl, JASPAR, TRANSFAC, and GeneCards can provide valuable insights and resources for researchers aiming to optimize AAV vectors for specific applications. By carefully selecting and designing promoters and enhancers, it is possible to improve the efficiency and specificity of gene delivery systems.

## 8 Multifunctional AAV Generation with AI

**Figure 3:**
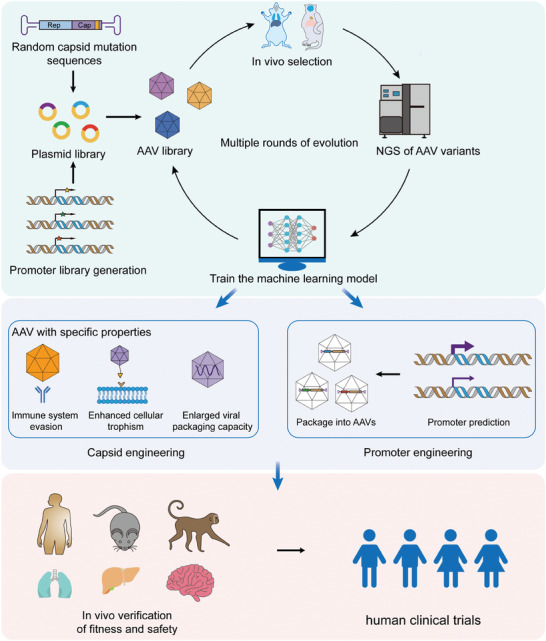
Machine learning‐guided AAV engineering strategies. Randomly diversified AAV libraries can be generated through in vivo evolution and subsequently sequenced using next‐generation sequencing (NGS) technologies. The resulting data can be utilized to train predictive models aimed at optimizing AAV promoters, tissue tropism, and other capsid properties.

The purpose of modifying the AAV vector through engineering techniques is mainly to enhance its safety and efficacy, but screening new AAV capsid variants and regulatory elements through experimental methods is time‐consuming and costly. Only a few studies have used AI to improve AAV characteristics such as yield, immunogenicity, distribution, and binding, and to optimize the promoters or enhancers (Table 1), and these studies have all focused on only one aspect of AAV vectors. Deep sequencing data and AI methods are currently being used in the development of a new generation of AAV vectors with the ability to avoid NAbs and that have enhanced tropism, increased packaging capacity, and enhanced promoters to meet the growing demand for gene therapies (**Figure** **3**).

Although AI methods provide powerful tools for optimizing viral vector design, there are still several limitations with these methods. First, the performance of the models greatly depends on the availability of large high‐quality datasets, but these are often difficult to obtain in the biomedical field. Second, many AI models, especially deep learning models, lack sufficient transparency and interpretability, which is crucial in the biopharmaceutical field where precise interpretation of model predictions is necessary to ensure biosafety. Additionally, models may be prone to overfitting, excelling on training data while underperforming on new data, which restricts their general applicability and reliability. Furthermore, the complexity of biological systems may exceed the current processing capabilities of AI models, which fail to fully capture and simulate all the critical factors in different biological processes. In addition, the high computational demands of advanced AI models may limit their use in resource‐scarce research institutions. Thus, despite the potential shown by AI technology in AAV capsid engineering, these technical and practical challenges need to be addressed before such methods can have widespread application.

## Conflict of Interest

The authors declare no conflict of interest.
